# Editorial: Microbiome in the first 1000 days: multi-omic interactions, physiological effects, and clinical implications

**DOI:** 10.3389/fcimb.2023.1242626

**Published:** 2023-06-30

**Authors:** Carla R. Taddei, Josef Neu

**Affiliations:** ^1^ School of Arts, Sciences and Humanity, University of São Paulo, São Paulo, Brazil; ^2^ Division of Clinical Laboratory, University Hospital - University of São Paulo, São Paulo, Brazil; ^3^ Division of Neonatology, University of Florida College of Medicine, Gainesville, FL, United States

**Keywords:** multi-omics approach, gut brain axis, infant´s microbiome, breast milk, first 1000 days, prematurity, diet and environment

The initial colonization of the human gut microbiota is of paramount importance and plays a key role in immune, nutritional, metabolic, and neurological development. With breastfeeding, the newborn is thought to receive beneficial intestinal bacteria, in addition to oligosaccharides, proteins, and immune cells, among other components, which aid in neonatal health and can prevent disease and sepsis (Carr et al., 2021). The first years of life are a window of acquisition and tolerance of the microbial community and its relationship to the infant’s immune system. Therefore, the expression “first 1000 days of life” has been proposed as a guideline for infant health care, from conception to the second year of life (Robertson et al., 2019).

During childhood, lack of breastfeeding, use of antibiotics, exposure to environmental contaminants, and poor nutrition select commensal members or pathobionts of the microbiota, to the detriment of symbionts. Thus, the presence of Gram-negative bacteria and the consequent unbalanced production of short-chain fatty acids (SCFAs) can lead to disruption of the intestinal barrier, increasing the intestinal permeability and tissue absorption of bacterial metabolites. These events are directly related to the local and systemic inflammatory response, with a consequent breakdown of systemic homeostasis and a significant impact on the child’s adult life, such as the manifestation of inflammatory and atopic diseases, diabetes, obesity, and allergies (Davis et al., 2022; Enav et al., 2022).

Similarly, preterm and low-birth-weight infants admitted to Neonatal Intensive Care Units (NICUs), are even more susceptible to environmental influences (Cuna et al., 2021). These babies are treated with antibiotics, prolonged parenteral nutrition (PN), and prolonged starvation and remain in the NICU for extended periods. These environmental factors associated with feeding difficulties reduce the colonization of the gut by symbiotic bacteria such as *Bifidobacterium* and *Lactobacillus*, which are important for the intestinal maturation process in infants, leading to abnormal colonization of the gastrointestinal tract (Saturio et al., 2021).

Since an aberrant microbiome community in early life has been associated with an increased risk of certain diseases, a better understanding of how the first 1000 days of life can influence the baby’s health process and how this might reflect on adult life is necessary. An integrative approach of multifactorial evidence in microbiome establishment must be explored, discussing the multi-omic interactions, physiological effects, and clinical implications. The aim of the present Research Topic was to discuss the multifactorial aspects that modulate the microbiome in the first 1000 days of a baby´s life and its possible outcomes and perspectives.

The role of pregnancy on the infant microbiome has been extensively discussed in the last years, and, despite evidence of the absence of viable bacteria in the fetus (Kennedy et al., 2021), there is still no consensus on intrauterine colonization. In addition, the role of prebiotic administration in pregnant women is still being discussed. In this sense, Cheng et al., described an animal model study using *Bifidobacterium bifidum* TMC3115 as a prebiotic for pregnant BALB/C mice. In an elegant experimental study, the authors analyzed the fecal microbiome of the offspring, cytokine levels in stomach contents, and transcriptome for the spleen and colon. The results showed a significant increase in cytokine levels and tissue expression in the treated animal group, in addition to immune modulation, suggesting a profound modulatory effect of the offspring microbiome by the maternal microbiome.

Indeed, the maternal microbiome seeding to the newborn seems to be multifactorial rather than vaginal site related. Dos Santos et al. conducted a longitudinal, prospective cohort study of 621 Canadian pregnant women and their newborns, comparing maternal vaginal samples collected pre-delivery and infant stools collected at 10 days and 3 months of age. Using cpn60-based amplicon sequencing, they found that the composition of the maternal vagina did not affect the composition and development of the infant microbiome, since the two communities were different, specifically at 3 months of age. Even though Dominguez-Bello et al., described the close relationship between the mode of delivery and infant microbiome (Dominguez-Bello et al., 2010), in this large cohort study, the authors demonstrated that the infant gut microbiome could be modulated over time by external factors, such as antibiotics and breastfeeding.

Discussing the role of pathological pregnancy in neonatal outcomes is even more challenging. In a preliminary study, Susic et al. showed changes in the gut microbiota of women with blood pressure disorders during pregnancy and their newborns compared to normotensive groups. In this study, the authors compared the gut microbiome of normotensive pregnant women (NP) and those who had a hypertensive pregnancy (HP), either gestational hypertension (GH) or pre-eclampsia (PE). Despite the small sample size, the authors described an enriched gut microbiome in the HP group, with *Bifidobacterium* and *Bifidobacterium* sp when compared to NP women, and altered gut microbiota of infants born to HP mothers, compared to infants born to NP women, corroborating the suggested association between the gut microbiome and pre-eclampsia, as previously reported (Chang et al., 2020). However, the collection of infant samples at 6 months after birth may have introduced confounding factors for the presented conclusions. Furthermore, the maternal microbiota throughout pregnancy needs to be considered to support a statement of this magnitude.

During childhood, modulation of the gut microbiome is associated with diet and environmental exposures (Enav et al., 2022), but individual genetic markers are already known to be determinants of health or disease outcomes, including microbiome development (Qin et al., 2022). Milletich et al. reported the importance of appropriate genotyping in addition to the interpretation of microbiome development. In their study, they were able to relate the association between celiac disease and the child’s microbiome through human leucocyte antigen (HLA) class II genotyping, which revealed three specific clusters in the microbiota of children with celiac disease: *Erysipelatoclostridium, Haemophilus* and the *Lacnospiracea* NK4A135 group, all of which are already related to inflammatory outcomes.

## Conclusions

New methodological approaches are changing the way we understand host-bacterial interactions. Studies evaluating the microbiome alone, such as those described above, are of interest but largely describe associations. In the future, we will need to integrate microbiome findings with other omics, such as the metabolome, proteome, and epigenome in order to establish mechanisms of action and provide stronger support for causality.

## Author contributions

All authors listed have made a substantial, direct, and intellectual contribution to the work and approved it for publication.
